# Viruses and the Brain—A Relationship Prone to Trouble

**DOI:** 10.3390/v17020203

**Published:** 2025-01-31

**Authors:** Matylda Barbara Mielcarska, Barry T. Rouse

**Affiliations:** 1 Department of Preclinical Sciences, Institute of Veterinary Sciences, Warsaw University of Life Sciences–SGGW, Jana Ciszewskiego 8, 02-786 Warsaw, Poland; 2 College of Veterinary Medicine, University of Tennessee, Knoxville, TN 37996, USA

**Keywords:** CNS, BBB, viral encephalitis, flaviviruses, Alzheimer’s disease, dementia, Parkinson’s disease, amyotrophic lateral sclerosis, multiple sclerosis

## Abstract

Neurological disorders, some of which are associated with viral infections, are growing due to the aging and expanding population. Despite strong defenses of the central nervous system, some viruses have evolved ways to breach them, which often result in dire consequences. In this review, we recount the various ways by which different viruses can enter the CNS, and we describe the consequences of such invasions. Consequences may manifest as acute disease, such as encephalitis, meningitis, or result in long-term effects, such as neuromuscular dysfunction, as occurs in poliomyelitis. We discuss evidence for viral involvement in the causation of well-known chronic neurodegenerative diseases, such as Alzheimer’s disease, Parkinson’s disease, amyotrophic lateral sclerosis, multiple sclerosis, as well as vascular dementia in the elderly. We also describe the approaches currently available to control a few of the neural viral infections. These include antivirals that are effective against human immunodeficiency virus and herpes simplex virus, as well as vaccines valuable for controlling rabies virus, poliomyelitis virus, and some flavivirus infections. There is an urgent need to better understand, at a molecular level, how viruses contribute to acute and, especially, chronic neurological diseases and to develop more precise and effective vaccines and therapies.

## 1. Introduction

Characteristically, the brain of vertebrates is highly protected from invasion by viruses and other factors that could damage its function. Indeed, getting into the brain is no easy task, and few exogenous viral agents can achieve success. There are two highly specialized multicellular obstacles that must be crossed by the virus present in tissues prior its entry to the brain. These are the blood–brain barrier (BBB) and the blood–cerebrospinal fluid (BCSF) barrier. By separating the bloodstream and cerebrospinal fluid (CSF) from the brain’s own cerebral extracellular fluid, the BBB and BCSF barriers secure the brain from invasion by viruses and other potential hazards such as foreign cells, toxins, and inflammatory factors [1,2]. Nevertheless, some viruses do succeed, and, when this happens, the consequences can be dire.

With some infections, severe damaging consequences rapidly occur, and these are highly disruptive to the brain’s function and are often terminal. Several insect-borne infections within the *Flaviviridae* family represent examples causing encephalitis [3]. However, some viral infections cause minimal overt damage, and the agents can persist in brain tissues for prolonged periods of time before being associated with lesions. One such example is amyotrophic lateral sclerosis (ALS), which is associated with an enterovirus that can invade motor neurons and maintain persistent infection in the central nervous system (CNS) [4]. In some viral infections, chronic sequelae can occur, which sometimes involve the immune system reactivity. Thus, viruses may trigger autoimmune lesions such as multiple sclerosis (MS), although its mechanisms are still unresolved. There is also evidence that viruses can participate in physical and mental decline, although associating these chronic consequences to direct effects of the infection is yet to be substantiated. For example, several viral types that achieve brain access, perhaps accidentally, could act as triggers for the initiation or acceleration of neurodegenerative syndromes such as Alzheimer’s disease (AD). Furthermore, there is now evidence that endogenous retroviruses, which represent up to 8% of the human genome, sometimes can be precipitated by environmental and perhaps other factors, can get involved in the etiopathogenesis of some inflammatory and neurodegenerative diseases (NDs), which include MS and ALS [5], as well as mental disorders. It is not uncommon to find the footprints of many different viruses in normal as well as diseased brain tissues, but linking the presence of these agents to the cause of either subtle or evident neurological events is highly problematic.

In this review, we describe the numerous mechanisms employed by viruses that permit entrance into the brain and mention the viral properties that can result in neuroinvasive disorders such as encephalitis, meningitis, as well as chronic damage to the brain. We also describe conditions where viruses are not overtly tissue damaging, but can persist in brain tissues and may act as primary or contributary causes of ND. These include AD, Parkinson’s disease (PD), MS, and ALS, together with vascular dementia (VaD), the second most common cause of dementia in the elderly people. During such evaluation, we comment on the value of current diagnostic and therapeutic procedures and suggest improvements that are still needed to clarify this confusing situation. Finally, we deal briefly with the contention that endogenous retroviruses may, in some instances, contribute to degenerative neurological diseases and that retrotransposons may affect motor neuron functioning. We take most of our viral-associated neuro-damaging examples from natural infections of humans and from animal models of infection, the latter of which can often reveal a misleading picture.

## 2. Getting into the Brain Is Not So Easy

**Figure 1:**
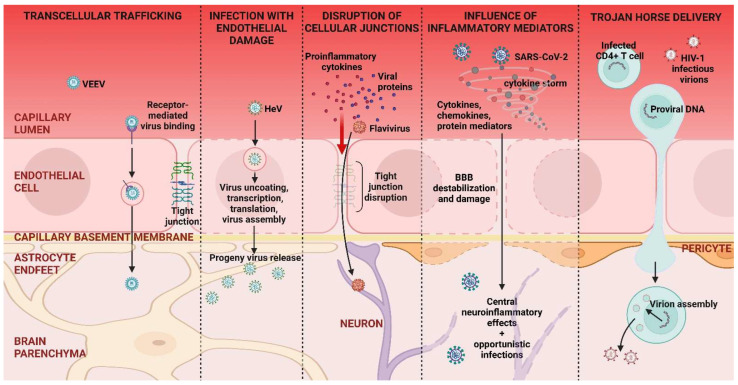
Mechanisms of virus entry into the central nervous system (CNS) through the blood–brain barrier (BBB). Viruses present in the systemic circulatory system can infiltrate the CNS by breaching the brain endothelial cells through various mechanisms. These include the following: transcellular trafficking without causing cellular damage; endothelial infection with damage; paracellular migration with the direct disruption of cellular junctions by viral proteins and with the participation of pro-inflammatory cytokines produced by activated barrier cells; disruption of the BBB with the involvement of pro-inflammatory cytokines and bioactive mediators that disrupt cell homeostasis, destabilize cellular junctions, and lead to the damage of cells composing the BBB; Trojan horse delivery within infected cells. For each of these mechanisms, viruses that exploit the pathway to infect the CNS are mentioned.

The brain is well protected against invasion, and few viruses have achieved success at gaining entry. Thus, in addition to the hard-to-penetrate bones of the skull, there are two highly specialized multicellular defense walls, the blood–brain barrier (BBB) and the blood–cerebrospinal fluid barrier (BCSF) that must be crossed if a virus or other factor such as a toxin or drug is to pass from a systemic location and enter brain tissue. The BBB is composed of non-fenestrated vessels comprising polarized endothelial cells connected by tight and adherent junctions, the capillary basement membrane, pericytes, and astrocyte endfeet, which provide an effective separation between blood and perivascular spaces [6] (see Figure 1).

The functions of endothelial cells, the central component of the BBB, are dependent on the interactions with pericytes and astrocytes as well as factors circulating in the CNS microenvironment such as cytokines and other soluble molecules [2,7].

**Figure 2:**
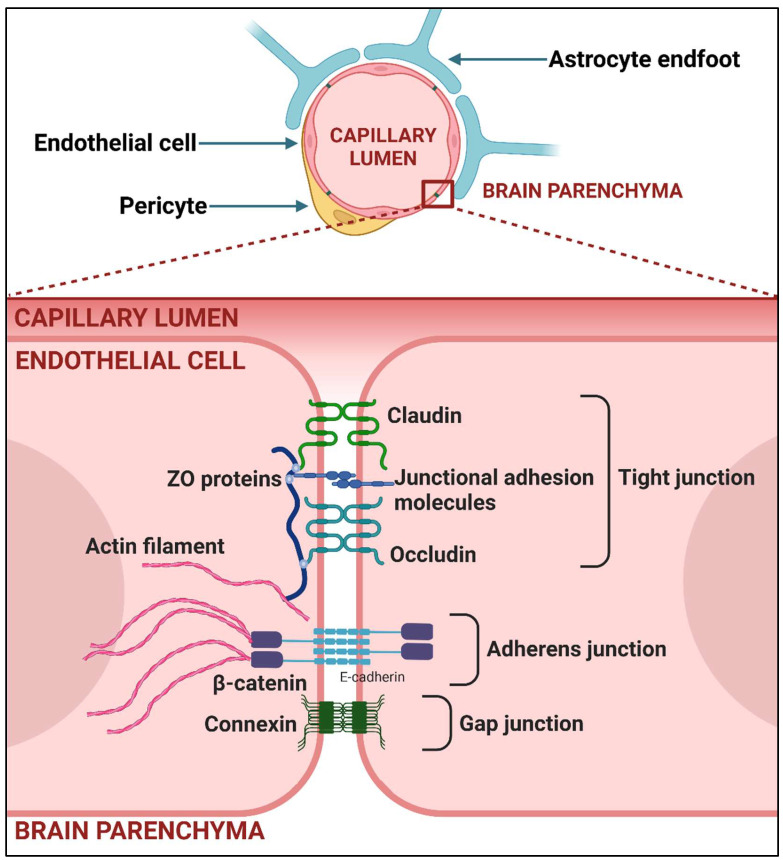
Intercellular junctions connecting the capillary endothelial cells of the blood–brain barrier. Tight junctions are formed by transmembrane proteins such as claudins, occludins, and junctional adhesion molecules, which are linked to cytoplasmic scaffolding zonula occludens (ZO) proteins that interact directly with actin filaments of the cytoskeleton. Adherens junctions are primarily composed of E-cadherins and β-catenins, the latter of which anchor the junctions to the actin cytoskeleton. Gap junctions are formed by two hemichannels in the plasma membranes of adjacent cells, which are composed of protein subunits called connexins.

One of the most prominent roles of the BBB is the precise regulation of paracellular, and thus vascular, permeability, which is attained with junction complexes that include tight, adherens, and gap junctions connecting endothelial cells [8,9]. Tight junctions (TJs) consist of integral membrane proteins such as occludins and claudins, with claudin-5 being the major constitutive claudin of the BBB. The TJ also comprise additional junctional adhesion molecules, cytoplasmic accessory zonula occludens phosphoproteins, and others [10]. The TJs provide low paracellular permeability to ions and small molecules and are major contributors to BBB properties [11]. The adherens junctions present a similar organization to the TJ and consist of cadherins and supporting catenins, and these regulate signaling processes as well as secure cell–cell adhesion [8]. Gap junctions consist of proteins from the connexin family [12], and play a role in cell proliferation and migration beyond intercellular communication [13]. The cytoskeleton linked to junction scaffolding proteins is also an indispensable component of intercellular connections. The types of junctions between BBB endothelial cells are depicted in Figure 2.

Vascular endothelial cells of the BBB are dynamic, semipermeable, and extremely selective and serve to regulate what can access and pass through the barrier. However, the BBB can be subjected to damage, and its permeability may increase as a person ages, accounting for the onset of age-related syndromes, some of which could be associated with opportunist viral infections, as discussed by Noe and colleagues [14]. Furthermore, some infections may accelerate NDs [15], and some have speculated that this happens in some survivors of COVID-19 [16].

The BCSF barrier is formed by a pair of membranes—one, composed of choroid plexus epithelial cells, and the second, the arachnoid membrane, composed of several layers of fibroblasts [17], that envelops the brain [18]. The choroid plexus-derived membrane consists of a layer of specialized ciliated glial cells that form the ependymal epithelium, which lines the ventricles of the brain and produces CSF on one side and surrounds capillaries on the other side. In comparison to the BBB, the BSCF barrier blood vessels are fenestrated, and astrocytes are not involved in the barrier structure. The BCSF barrier is a critical determinant of brain homeostasis and has several functions. These include secreting and regulating the ionic and nutrient composition of CSF, which provides a route for signal transduction between the choroid plexus and the brain via CSF [19]. This also limits the access of potentially neurotoxic compounds to the CNS [20]. Moreover, the BCSF barrier participates in the recruitment, skewing, and suppression of immune cells from the bloodstream into the CNS [21]. The BCSF likely prevents most viruses from accessing the brain, although how this is accomplished has received minimal investigation in comparison to the BBB. Several BCSF barrier models have now been developed so further understanding of the consequences of virus–BCSF interaction is anticipated [22]. Other recent studies indicate that aging is associated with morphological and molecular changes in the BCSF barrier, as well as altered CSF production and turnover. These changes may contribute to age-related cognitive decline and neurodegenerative disorders and perhaps to susceptibility to viruses that could help cause some of these lesions [21,23].

The protection of the CNS has been suggested to reflect the defense of a medieval castle [24,25]. The castle is surrounded by two walls bordering a moat, located behind a closed gate, hidden, but in plain sight [26,27]. However, despite these protective barriers, castles like the brain were never invincible, and some viruses do achieve neuroinvasion. We next describe the different strategies that viruses employ to achieve this maneuver.

## 3. What Makes a Virus Capable of Neuroinvasion?

There are basically two main avenues for a virus to enter the undamaged CNS: passage by way of nerve axons that synapse in the CNS or traversing the protective BBB and BCSF barriers. Those human infecting viral pathogens that take the former route include rabies virus (RABV), poliovirus (PV), and alpha herpesviruses. Curiously, with the latter virus infections, when the agent is not a specific pathogen of the species, oftentimes the virus transports to the brain to cause lethal encephalitis, which is an uncommon outcome in the natural host. An example is human infection with Herpes B, an insignificant pathogen in some primate species [28] but often causing lethal encephalitis if humans are infected. Moreover, although, immunologically, normal humans rarely suffer encephalitis following herpes simplex virus (HSV) infection, some primate relatives infected with HSV are highly prone to develop encephalitis [29]. A similar example occurs with alphaviruses in some domestic animals [30]. Thus, Pseudorabies virus (PRV) usually causes a peripheral infection in its natural host, the pig, but it is apt to result in encephalitis if cattle, feline, or canine species are infected [31,32]. The reasons for these differences in viral tropism are not fully understood, but herpesviruses always establish a second lifestyle in animals they infect, referred to as latency. With alpha herpesviruses, latency is established and maintained in the peripheral nervous system but is subject to reversal from time to time. When this occurs, the pathogen commonly passes anterogradely via sensory nerves to peripheral sites where recurrent lesions may occur [33]. During reactivation, the pathogen may also disseminate via neuronal projections from the latency site to the CNS, causing severe encephalitis or raising no symptoms, but the frequency of the latter phenomenon occurrence remains unknown. There is some evidence that HSV can persist in the CNS and perhaps be a cause of neurodegenerative disorders, although this remains an area of controversy [34]. There is also evidence that some of the flaviviruses may access the CNS by infecting olfactory neurons or peripheral nerves and exploit retrograde axonal transport [35,36], although it is not known how often this route occurs compared to crossing the BBB.

### 3.1. Neuronal Route of Infection

An example of a virus using the axonal transport route to invade the CNS is RABV, and this usually ends in fatal encephalomyelitis [37]. After inoculation by a bite or scratch from an infected animal, the virus enters a motor neuron of the PNS via receptor-mediated endocytosis at the neuromuscular junction [38,39]. RABV retrogradely traverses the neuron by hijacking the microtubule transport machinery [40,41]. After reaching the cell soma, the virus replicates and spreads trans-synaptically to subsequent neurons to ultimately disseminate in the CNS to culminate in death of the host [42]. Once in the CNS, the virus encounters scanty obstacles to its spread since neuronal cells have few, if any, immune defenses, and the virus produces multifunctional proteins that further inhibit the extent of any innate immune reactions, for example, RABV P [43,44]. This occurs by mechanisms that include the production of proteins that arrest interferon signaling, inhibit inflammatory cell infiltration to the CNS, and induce apoptosis in infiltrating T cells [45,46].

Once within neurons, RABV induces major alterations in gene expression [47], in axon and dendrite degeneration [48,49], as well as neurotransmitter dysregulation [37,50]. Interestingly, in some instances, the brain of RABV-infected animals contains a small number of necrotic neurons without evidence of inflammation, yet animals still suffer from lethal consequences [51,52]. This likely happens because the field strain of RABV primarily infects and damages astrocytes, with neuronal dysfunction as a secondary effect of astrocyte infection. In fact, highly virulent field RABV strains exhibit high tropism for non-neuronal cells in the CNS, with astrocytic infection frequencies far exceeding neuron infection [53,54].

Alpha herpesviruses can also invade the CNS via the neuronal route and exploit the nerve axon transport system. In the case of HSV-1, cellular entry requires the use of specific cellular receptors, which is a key factor determining tissue and cell-specific susceptibility to virus infection. Viral nucleocapsids traffic along microtubules to reach the trigeminal ganglia (TG) and the brainstem by afferent inputs from the TG, where latency is established [55]. Occasionally, primary HSV infection or reactivation from latency may turn into severe acute inflammation, resulting in potentially fatal necrotizing meningoencephalitis [56,57]. Although a rare disorder, herpes simplex encephalitis (HSE), is the most common sporadic infectious encephalitis in humans [58]. One of the determinants of HSV neurovirulence includes the extent of the type I IFN response that limits virus replication, spread, and cytopathic effect in the brain [59]. Mutations that reduce the CNS-intrinsic IFN production in response to HSV-1 may predispose to HSE [60,61]. A comparison of two different viruses using retrograde axonal transport to reach the CNS: RABV and HSV-1 is summarized in Table 1.

In the past decades, a frequent cause of neurological lesions was poliomyelitis virus, (PV), now almost eliminated by effective vaccines and the WHO eradication and endgame program. PV infections are mostly asymptomatic, with viral replication largely confined to the tissues of the oropharynx and gastrointestinal tract, typically accompanied with a brief period of viremia [70,71]. However, in some individuals, the circulating virus may access the CNS either via the BBB or be retrogradely transported along the axons of peripheral nerves. In the latter process, virions undergo endocytosis at synapses, and virus-containing endosomes are transported along the axon, from peripheral regions to the neuronal cell body [72]. Once there, viral particles are uncoated and virus replicates. Interestingly, the pathway of PV dissemination depends on the virus strain [73]. CNS invasion by PV can lead to non-paralytic, aseptic meningitis in a small proportion of cases, or to the infectious destruction of alpha motor neurons located in the anterior horns of the spinal cord, as well as cranial nerve nuclei in the medulla oblongata, pons, and midbrain, resulting in characteristic paralytic poliomyelitis [71]. Surprisingly, most sensory neurons and much of the brain, aside from the brainstem, remain unaffected during the disease. The pattern of viral localization may be attributed to the intrinsic differences in the susceptibility of various neuronal cell types, host genetic variants in the innate immune defenses and cell death pathways, as well as and possibly the route of viral spread [73,74]. The relationship between the latter and the disease’s clinical form, severity, and eventual pattern of paralysis awaits to be determined.

Another route of neuroinvasion that bypasses the CNS barrier defenses is anterograde transport through olfactory sensory neurons. Such cells have their cell bodies in the olfactory epithelium, and their axons pass from the nasal to the cranial cavity to terminate in the olfactory bulb (OB). This makes them the only CNS cells exposed directly to the external environment [51,75]. Various DNA and RNA viruses are initially detected within the OB during neuroinvasive infection, which include herpesviruses, some neurotropic flaviviruses, measles virus (MeV), influenza A virus, and others, as reviewed by Durrant et al. [75].

Interestingly, retinal cells of the eye were recently suggested as another entry point for virus neuroinvasion [76]. Severe acute respiratory syndrome coronavirus 2 (SARS-CoV-2) can exhibit ocular tropism, permissively infect, and therefore replicate in retinal cells in vitro [77]. However, if and how the virus may spread into adjacent neurons, such as the neighboring optic nerve constituting the extension of the retina, remains to be clarified.

### 3.2. Hematogenous Route of Infection

The more common route of entry for neurotropic viruses, as well as for most accidental viral invaders, is from the bloodstream and crossing either the BBB or BCSF barriers. As mentioned before, in normal circumstances, these barriers are difficult to transverse as the membrane cells are tightly attached to each other, and the defense of the site is assisted also by innate immune activities. Those viruses that do succeed have developed a number of strategies. Many of these are listed in Table 2, along with examples of viruses that use the specific strategy.

The mechanisms used to cross the barriers vary, with some viruses passing with no or minimal damage to the barrier cells and junctions between them, whereas others pass by, causing notable damage. Some viruses are only able to pass if the barriers are made more permeable, such as by exposure to pro-inflammatory factors and mediators. The increase in barrier permeability can also occur under the sole influence of some proteins derived from viruses themselves. Finally, passage can be achieved by the so-called Trojan horse mechanism, which is a combination process that we further describe in a separate section.

#### 3.2.1. Neuroinvasion from the Blood Stream

The most prevalent agents that can enter and infect brain cells are insect-transmitted members of the Flavivirus and Togavirus families, which initiate the infection at an extraneural site. Many of these viruses achieve passage into the CNS by the binding of a surface viral glycoprotein to a receptor on BBB endothelial cells, after which cell entry occurs by exploiting clathrin-coated or caveolae-mediated endocytosis. The virus is then released into the CNS tissue without causing overt damage to the endothelial layer. Traversing BBB may occur with or without replication [78], and, once in the CNS, flaviviruses primarily target differentiated neurons [79,80] but also can infect neural progenitor cells (NPCs) [81], astrocytes, and microglia [82,83]. Research on WNV, JEV, TBEV, and ZIKV in vitro and ZIKV-infected animal models has also demonstrated that the transcytotic crossing of endothelial cells caused no changes in BBB overall permeability or integrity [84,85,86,87,88]. Similarly, other arboviruses, such as Venezuelan and Western equine encephalitis viruses (VEEV and WEEV, respectively) from the *Togaviridae* family, transcytose through brain microvascular endothelial cells without damaging the BBB, as shown by in vitro studies with BBB cultures [89]. However, further research is necessary to verify if these findings hold true in vivo in humans.

A different scenario ensues when viruses that encounter the barrier cells infect and damage the barrier function, and this is often followed by wide scale invasion of the CNS. This situation happens with *Paramyxoviridae* pathogens such as Hendra virus (HeV) and Nipah virus (NiV) infections [90]. The major characteristic of infections by HeV or NiV is an acute, severe respiratory illness and a widespread endothelial infection in the CNS that rapidly progresses to an often fatal encephalitis [90]. With both pathogens, after a respiratory phase, viremia occurs, and the virus in free form, or bound to host leukocytes, makes contact with BBB endothelial cells as well as choroid plexus blood vessels [91,92]. Cell fusion and syncytium formation mediated by viral envelope glycoproteins, e.g., Nipah virus F envelope glycoprotein (NiF) and NiG then occur, and this results in extensive damage and ready access to cells in the CNS [93]. At the same time, an excessive induction of inflammatory cytokines may occur that further facilitates access to brain tissues [94,95].

In some instances, viral proteins alone also have the potential to substantially disrupt the barrier structure. This can occur with Human Immunodeficiency Virus (HIV-1) and some flavivirus infections [96,97,98]. In the case of HIV-1, the Tat, gp120, and Nef proteins can damage the BBB, causing apoptosis and oxidative stress as well as changing the adhesiveness of endothelial cells [99,100,101]. These proteins also hijack membrane trafficking to degrade or displace host antiviral proteins and activate cytokine secretion, all of which can profoundly affect the endothelium and other BBB components [102,103]. In addition, some flavivirus proteins also have been linked to causing increased endothelial permeability, as detected by some in vitro and in vivo studies [104,105]. For example, the nonstructural protein 1 (NS1) from DENV, ZIKV, WNV, and JEV can bind to the endothelial glycocalyx layer, disrupting its integrity and inducing breakdown [106], as well as contributing to endothelial cells’ apoptosis or lysis [107]. When injected into animals, the DENV NS1 alone can induce the production of key inflammatory cytokines, leading to brain endothelium dysfunction [108,109]. Other flavivirus nonstructural proteins, such as NS2A and NS2B, were shown to act as viroporins and alter membrane permeability in different models [110,111]. However, their role in crossing brain barriers in vivo in human systems awaits exploration [112].

Other viruses that infect the brain mediate the changes they cause indirectly by inducing various cytokines, chemokines, and other protein mediators that in turn increase their access to the brain. Such effects have been observed with some flaviviruses, HIV-1, SARS-CoV-2, and a few other infections [113,114,115]. The effects of these mediators set the scene for the disease pattern that follows [116]. In some cases, induced factors, such as interferons, can act to minimize or even terminate the infection. However, other mediators function to increase the permeability of the brain barriers and hence increase the likelihood of spreading infection, and additional mediators may contribute to the severity of damage once the barrier is crossed [83,96,117]. In some conditions, the virus may not enter the CNS, but central neuroinflammatory effects still occur, likely attributed to mediators. This scenario has been advocated to occur occasionally when SARS-CoV-2 infection results in a “cytokine storm”, a state of hyperinflammation that may lead to neurological lesions [118,119,120]. Pro-inflammatory cytokines and other mediators released during this phenomenon activate barrier endothelial cells and astrocytes and further microglia. These effects induce and amplify neuroinflammation and destabilize and damage the BBB [120,121]. Finally, at times, the induction of brain-damaging mediators has been attributed to the effects of viral components inducing them within the CNS rather than the result of actual CNS infection [104,122]. This may be occurring in some cases of neural problems associated with HIV and perhaps DENV infections [123].

Among the pro-inflammatory mediators that may weaken guard barriers, the tumor necrosis factor (TNF) and interleukin (IL)-1β are the most prominent. In vitro studies have shown these to alter the organization of junctional complexes and transcellular trafficking [124]. In addition, IL-6 reduces the expression of a variety of intercellular junction proteins [125], and interferon-γ (IFN-γ), secreted by activated leukocytes, can cause the redistribution of TJ and AJ proteins in culture systems. Additionally, high IFN-γ and TNF concentrations may induce endothelial apoptosis [126], and elevated plasma IL-8 and transforming growth factor-β (TGF-β) levels have been advocated to be associated with BBB disruption and increased BBB permeability [118,123,127]. All these pro-inflammatory effects may be counterbalanced, to a varying extent, by the inhibitory activity of the type I IFN response that the viruses induce. Thus, the overall outcome of neural infection by virus is hard to predict in individual infections, and far more studies are warranted, especially using human systems.

#### 3.2.2. Trojan Horse Delivery

The intriguing Trojan horse idea that a virus might succeed in entering the brain hidden within cells that can cross barriers was proposed by Ashley Haase and Bill Narayan to explain how the lentivirus Visna achieved brain access [128,129]. This idea was later extended to explain how mouse cytomegalovirus gets into the brain, and it has been advocated too as a major way that HIV-1 and HCMV gain brain access [130,131,132]. The essence of the Trojan horse concept is that the virus can only access the brain within a cell such as a monocyte, dendritic cell, or lymphocyte, which remains unchanged and may be endowed with heightened mobility and other activities to achieve such passage. Another required step may be that the barrier itself usually needs to be made more receptive by exposure to inflammatory cytokines and chemokines such as the TNF and/or IL-1β, and monocyte chemoattractant protein-1. The Trojan horse idea has been pursued largely by in vitro studies that have focused particularly on HIV-1, HCMV, and, more recently, SARS-CoV-2 [133]. However, proving that the mechanism is a major brain access portal in vivo is problematic, and how such knowledge can influence the selection of protective therapies needs be explored.

#### 3.2.3. Hematogenous Route of Neuroinvasion, Conclusions

In summary, from using in vitro models and some animal models of virus infection, numerous routes have been identified by which a virus can pass from the bloodstream to infect the cells of the CNS [134,135]. Well-studied flaviviruses, such as WNV, can penetrate the CNS through multiple mechanisms, and almost certainly there could be several alternative ways for any particular virus to achieve entry. Furthermore, the pathway employed could vary, depending on the circumstances of infection, genetics of the host, and the past disease history [136]. Sorting out what actually occurs could be highly relevant, especially if the therapies available were more effective against one mechanism than against another. Alas at present, successfully preventing and controlling most acute and particularly chronic viral-associated neurological diseases is not usually a practical procedure, but this scenario is likely to be clarified by further studies.

## 4. Once the Barriers Protecting the CNS Have Been Breached

### 4.1. The Disease Scenario When Viruses Infect the Human Brain

Should they succeed in gaining entrance to the CNS, several types of viruses cause neural damage. We have already described some examples in the previous sections, and Table 3 lists the main named viruses that, in humans, cause neuropathologies of clinical consequences.

The outcome of neural invasion can occur quickly after infection, as happens with several flaviviruses, or may take a prolonged time period (up to years) to become manifested. In the latter scenarios, the role of the virus infection, especially as a sole cause of the clinical syndromes, is debatable, as will be mentioned in the sections describing those diseases. The CNS infection outcome is closely linked to the extent of cellular damage that it induces. Already during the viral crossing of the CNS barriers, immune and barrier endothelial cells can undergo activation and release pro-inflammatory factors, which in turn can lead to reactive astrogliosis and microglial activation [198]. Inflammatory cytokines, mediators, and neurotransmitters released by activated glial cells may potentiate the neuroinflammatory state and lead to neurotoxicity and ultimate cell death. CNS cell death may occur via apoptosis; for example, viral proteins may disrupt the equilibrium of various Bcl-2 (B-cell lymphoma 2) family members, leading to the execution of this death pathway. Inflammation can elicit cell death by necroptosis or pyroptosis, mediated by the activation of the TNF receptor or receptors and proteins recognizing viral PAMPs, or by different inflammasome pathways, respectively [199]. Necrotic cells or focal necrotic lesions may also be observed, where severe cellular damage was irreparable, and cells died as a result of necrosis [200].

Perhaps the most famous infectious agents that cause damage to the CNS are prions, which lack nucleic acids but are self-replicating proteins. These are chronic, usually lethal infections that primarily damage the CNS. Examples in humans are Kuru, Creutzfeldt–Jakob syndrome, and, occasionally, infections transmitted from prion-infected animals such as bovine spongiform encephalopathy, also called “mad cow disease”, chronic wasting disease affecting cervids throughout North America, and scrapie affecting sheep and goats [201]. Finally, there is evidence that, under some circumstances, endogenous retroviruses, which account for up to 8% of the human genome, may become activated and act as, at least, a cofactor in the expression of some neurological syndromes. This issue is briefly discussed in a later section. The descriptions that follow cover the agents that can cause mainly acute damage to the CNS and that were not described in the section on how viruses enter the CNS.

#### 4.1.1. Flavivirus Infections of the CNS

Several members of the insect-transmitted Flavivirus family can invade the CNS in humans and also domestic animals, leading to severe and sometimes long-lasting neurological disorders and even to death. However, most infections caused by these viruses in humans are asymptomatic or result in mild clinical signs often confused with influenza, but some become febrile and develop acute neurological signs that can progress to encephalitis, and some infected patients suffer long-term neurological complications and lasting disabilities. The outcome of infection with four of the most common flavivirus agents that affect humans is summarized in Table 4.

Not all members of the *Flaviviridae* family are regular causes of neurological problems, with hepatitis C virus (HCV), YFV, and dengue viruses as notable examples. In addition, some, such as ZIKV, are primarily neural pathogens when they infect the fetus or newborn. Additionally, some flavivirus infections have been associated with subsequent chronic syndromes, such as autoimmune-related disorders and systemic or metabolic disturbances, which may contribute to neurological outcomes.

In order to cause neural lesions, flaviviruses must pass from the blood and enter the CNS, which requires passage across the BBB. Once within the CNS, the outcome depends on the cell type, location, and extent of the infection and, in some instances, the ability of the virus to manage the extent of the host response to infection. JEV, WNV, and TBEV are considered to cause some of the most severe neuroinfections and can present as acute and fulminant encephalitic syndromes with a high mortality rate (see Table 3). Among them, JEV causes severe neurological symptoms, and they are followed by a greater loss of disability-adjusted life years than any other virus transmitted by arthropods [208]. Flaviviral acute encephalitis is accompanied by high viral titers within the brain, elevated levels of inflammatory cytokines and chemokines, disruption of the BBB, and other vascular damage, resulting in increased intracranial pressure and significant swelling in the CNS [235]. Additionally, lesions and necrotic areas are observed in various brain regions, with the specific locations depending on the flavivirus involved [236]. Patients may present behavioral and movement abnormalities, spastic paralysis, and cognitive disorders. Many survivors of the acute illness develop post-encephalitic syndrome and/or suffer from a wide array of long-term or permanent neurological sequelae involving the CNS, PNS, or both [237,238]. JEV, in particular, has the potential to recur months after primary acute encephalitis [239].

With flavivirus infections, neurons are the primary target cell type infected, and such cells may be killed directly by the virus or damaged indirectly by immune-mediated mechanisms. Non-neuronal cells susceptible to infection by selected flaviviruses are listed in Table 5.

The question of whether WNV infects microglia remains contentious, as these cells fail to support viral replication [242]. Glial cells, BMECs, and even some inflammatory cells may serve as potential reservoirs for flaviviruses, allowing for prolonged viral persistence within the host [252,253,254,255] and contributing to the nature of the clinical signs. There is evidence that viral RNA and perhaps complete virions can remain in the CNS for extended periods of time [202,256,257]. In fact, the diseases caused by flaviviruses can present with a wide spectrum of consequences. This likely reflects the distinct cellular and tissue tropism of each virus, variations in their ability to evade or counteract host immunity, and the interplay between pathogenic effects of infection and damage resulting from the host response [3,258]. Indeed, we need to fully understand what factors account for the variable outcome of flavivirus infections with a view to provide useful clues to control these troublesome agents that are of increasing relevance in many locations [259,260,261].

Evidence accumulates showing that some flavivirus infections could be involved either as causes or at least cofactors in the outcome of some chronic neurodegenerative disorders. Some of these disorders are considered primarily as autoimmune lesions (see Table 4 and Table 6).

For example, approximately 45% of Japanese encephalitis survivors suffer from Parkinsonism [274], the term encompassing multiple neurodegenerative disorders characterized by motor dysfunction and unstable posture [275]. In addition, chronic tick-borne encephalitis can present with a number of neurological conditions, which include a disease resembling PD [276], and survivors of WNV infection can experience depression, memory loss, as well as motor dysfunction [277]. Systemic DENV infection that affects the neuroaxis is also indicated as a potential risk factor for developing NDs [278]. Notably, within the *Flaviviridae* family, DENV accounts for the highest number of annual infections, estimated at 100–400 million cases worldwide, with up to 20% of these cases considered to involve the CNS [279,280]. Abundant studies using animal models of flavivirus infections also have revealed similarities between persistent viral lesions and changes observed in AD, PD, Huntington’s disease (HD), or multiple sclerosis (MS) [277,281]. Even if the solid evidence that flaviviruses are the causes of chronic neurodegenerative diseases in humans is not available, their acting as cofactors in some cases would seem a likely event. Moreover, in situations where a flavivirus is implicated as a cause of either acute or chronic neurodegenerative syndrome, there is the possibility of control by using vaccines. Successful vaccines are available for several flaviviruses, especially to control animal diseases.

#### 4.1.2. HIV Infection of the CNS

HIV primarily targets helper T-lymphocytes and macrophages that express the CD4 molecule, the main cellular receptor for this virus [282]. As early as 1–2 weeks after the primary infection, the virus can gain access to the CNS and is transported there mainly by being present within transmigrating cells, such as monocytes, macrophages, and T-lymphocytes, the often-called Trojan horse infection mechanism, or on the surface of DCs [283,284,285]. The early neuroinvasion by HIV triggers inflammation in the brain, but neurological symptoms are scarce, and only 1–4% of patients develop acute neurological disease. The HIV-infected cells accumulate within the brain parenchyma, where the virus replicates and is released, leading to infection of other CNS cells. The virus may persist in the brain throughout the individual’s lifetime [286] and, in the early phase, this occurs mainly in T cell populations. However, over time, the virus targets perivascular macrophages and microglia, which become the primary sites for HIV replication [287,288,289]. The virus also infects astrocytes, of which up to 20% can harbor a DNA copy of the HIV RNA genome, as they often bear latent and nonproductive viruses [290,291]. Interestingly, HIV-infected macrophages, microglia, and astrocytes are unlikely to undergo apoptosis and can maintain viral infection despite antiretroviral therapy (ART) [195,292,293]. This likely contributes to virus persistence in the CNS [294]. The presence of a viral reservoir in the CNS is recognized as a major challenge to eradicating HIV using ART [295].

Although HIV does not directly infect neurons, infected individuals experience neuronal injuries, such as synaptodendritic damage, the dysregulation of myelination, and axonal damage. The ensuing neuronal loss may result in cognitive difficulties [294,296]. However, the actual causes of neuronal damage and loss are multifaceted and can be attributed in part to the production of neurotoxic viral proteins derived from productively infected non-neuronal cells but, in some cases, even from latently infected cells following low-level transcription [297]. The other cause of neuronal damage is a bystander consequence of an immune reaction triggered in response to viral replication or viral persistence in brain cells [298,299].

Over the course of infection in non-neuronal brain cells, various HIV-encoded proteins may be released that can have a range of effects on nearby cells that include the direct toxic damage of neurons (see Table 7).

Several HIV proteins mediate such paracrine effects, with Tat, gp120, and Nef the most prominent. Additionally, these proteins can induce the expression of various pro-inflammatory cytokines in neurons or activate other types of CNS resident cells [122,304,307]. HIV proteins exhibit both proapoptotic and antiapoptotic properties [321,322] and alter intracellular ionic homeostasis, entailing dysregulation, injury, and death of neurons or non-neuronal cells [326,327,328]. These effects may result in excitotoxic damage to neurons, potentially contributing to neurodegenerative processes [346]. Additional changes include inflammatory cytokine induction, changes in cell-to-cell communication [334], migration patterns, and the propagation of toxic signals to neighboring cells [359]. This functional diversity underscores the critical role of HIV proteins in the development of HIV-associated neurocognitive disorders.

During CNS invasion, HIV-infected monocytes present in the CNS, along with macrophages, microglia, and astrocytes, release inflammatory mediators such as the TNF, IL-1β, IL-6, IFN-γ, IL-8, CCL2, CCL5, CXCL10, as well as the platelet-activating factor (PAF), nitric oxide, matrix metalloproteases (MMPs), and arachidonic acid [294,360]. These factors disrupt neuronal homeostasis and metabolism, causing neuronal injury and BBB damage, allowing a greater leakage of serum proteins and other substances into the brain. Additionally, the release of pro-inflammatory factors in the brain of patients with neural signs was shown to dysregulate levels of neurogranin, a protein essential for maintaining synaptic integrity and function [361]. Furthermore, the pro-inflammatory cytokines activate uninfected cells and facilitate the recruitment of infected cells from the bloodstream to the CNS [362]. This exacerbates the cascade of neuroinflammation triggered originally by a small number of transmigrated cells [294]. These consequences of HIV brain infection are highly variable both in terms of the damaging events that occur, their location within the CNS, and the time after infection that lesions become clinically significant.

In high-income countries, most patients diagnosed as HIV-infected are treated with antiviral drugs, and these can be extraordinarily effective at controlling acute disease manifestations, but none succeed in removing the virus from the body, especially when the virus is present in the brain. Many patients infected with HIV eventually develop neurological problems, and these are less prevalent in patients treated with ART [363].

The most common neurological outcome is termed HIV-associated neurocognitive disorders (HANDs), and this has dropped from affecting about 20% prior to ART to around 2–5% today [364]. While treatment reduces neuronal breakdown, patients on therapy continue to experience ongoing direct virus-related damage or aging-like repercussions attributable to HIV infection [365]. Notably, even in the ART era, the virus persists within the brain, and this could contribute, along with other comorbidities such as age, diabetes, hypertension, and hypercholesterolemia, to the onset of cognitive impairments that often occur in HIV patients [366]. As individuals with HIV age, they face increasing risk of the accelerated onset of other neurodegenerative disorders, particularly AD [367,368,369,370], AD precursor, amnestic mild cognitive impairment (aMCI) [371], Parkinsonism [372,373,374,375,376], vascular dementia [297,377], rarely ALS-like symptoms [378,379], and others. The topic of HIV is complex and fascinating but has received some excellent reviews worth consulting for details [380,381,382,383,384].

#### 4.1.3. SARS-CoV-2 Infection of the CNS

The SARS-CoV-2 pandemic has profoundly affected human health and the public’s opinions about the value of vaccines. The virus can infect many locations, with the occurrence of encephalitis as a complication in severely ill COVID-19 patients, around 7% [385,386]. Many survivors, including those with mild symptoms, continue to suffer from a debilitating post-viral condition known as LC/PASC (long COVID/post-acute sequelae of SARS-CoV-2) [387]. PASC may share features with myalgic encephalomyelitis and chronic fatigue syndrome. It includes cognitive deficits, the loss of smell, muscle pain, and autonomic dysregulation, as reviewed by Pandya [388]. The potential factors contributing to PASC are viral persistence, activation of the immune responses and autoimmune processes [389]. These consequences are potentially involved in long-term neurological disorders that appear after SARS-CoV-2 infection. Although SARS-CoV-2 is associated with acute encephalitis and neurological signs in some patients with LC, whether such signs result from a replicating virus present in the brain is uncertain. However, SARS-CoV-2 antigens and RNA were reported post-mortem in the brain parenchyma of some victims [390,391], but the findings have not been widely supported. Suggestive features such as neuroinflammation, gliosis, vascular, and BBB damage were observed in the brains of some COVID-19 patients [392,393], and others reported extensive neuronal degeneration and morphological changes in glial cells, particularly in the hippocampus of affected individuals [394].

It is conceivable that the neurodegenerative consequences of SARS-CoV-2 could be an indirect result of infection, such as the induction of a cytokine storm that occurs in some patients [395]. In fact, the occurrence of cytokines and chemokines in the CSF, as well as in the circulation, has been documented [396]. Currently, we must conclude that evidence linking SARS-CoV-2 infection directly or indirectly to neurodegeneration remains equivocal and whether SARS-CoV-2 infection alone is sufficient to initiate neurodegeneration remains to be firmly established, as should happen with further research.

### 4.2. Chronic Neurodegenerative Diseases and Viral Infections

The burden of disability and deaths due to neurological disorders is increasingly recognized as a global health challenge [397]. Neurological disorders are the main cause of human physical and cognitive disability worldwide and the second leading cause of death according to a study analyzing data from 195 countries over 30 years [398]. The burden of NDs, a neurological disorder subset, is expected to at least double in the coming twenty years, giving rise to a significant challenge to keep neurological care accessible to everyone [399]. NDs are diverse in pathophysiology, and multiple risk factors underlie their development, such as genetic makeup, environment, infections, as well as age, which appears to be the most consistent determinant [400].

Senescence entails a multitude of changes across the spectrum of body systems [401], and the brain deteriorates with age in parallel with other organs. Aging causes a decline in cognitive functioning, which is related to brain atrophy and significant changes to the brain’s vascular structure and function [402,403] at varying extents in different brain regions. Further, with increasing age, innate and adaptive immune systems undergo senescence, and increasing inflammatory functions entail chronic low-grade inflammation that could contribute to ND [404]. NDs have increased rapidly in incidence in recent years along with the expansion and aging of the human population [405] and are a common cause of cognitive impairment and morbidity in older people [406].

There are numerous chronic neurodegenerative syndromes, many given a name where one of the likely causes could be a virus infection. In this section, we describe many of these focusing on the role that a virus infection might be playing in their cause and expression.

#### 4.2.1. Parkinson’s Disease (PD)

PD is primarily a disease of older people. It affects approximately 1–2% of individuals over 65 and increases to 4–5% in those reaching 85 [407]. While genetic susceptibility factors explain 10–20% of PD cases, the vast majority have no clear underlying cause identified. The disease is marked by the presence of abnormal intracellular aggregates known as Lewy bodies, Lewy neurites, and cytoplasmic inclusions in glial cells that are primarily composed of α-synuclein proteins [408]. Key pathological features of PD include progressive degeneration and the loss of dopaminergic neurons, particularly in the *substantia nigra pars compacta* of the midbrain, basal ganglia, and other brain regions, along with a corresponding reduction in dopamine in the striatum [409]. The PD clinical signs encompass motor features such as resting tremors, postural instability, bradykinesia and muscle rigidity, along with non-motor symptoms such as cognitive decline and dementia [410]. Disease manifestations arise when approximately 60% of neurons within the nigrostriatal pathway die [411]. Without the Lewy bodies and other PD pathological hallmarks that characterize primary PD, secondary Parkinsonism may appear, which is similar in clinical presentation [412]. An unresolved question is do virus infections either help set off or contribute to the pathogenesis of PD? Several clues suggest this could occur.

For example, following the 1918 H1N1 influenza A pandemic, cases of post-encephalitic Parkinsonism became a more common occurrence [413], but later H1N1 flu pandemics did not find a similar association, and the virus was not detected in brain of the victims. Moreover, the infection of mice with neurogenic strains of influenza mimics PD [414]. By meta-analysis, infections with HSV, MuV, HBV, and HCV have been associated with an increased risk of PD [415,416], and successful HCV drug therapy was associated with a reduced risk of subsequent PD [415,417]. Infections with Saint Louis encephalitis virus, JEV, WNV, Coxsackie virus and HIV can all produce some core motor symptoms characteristic of PD and were associated with postencephalitic Parkinsonism [418]. Other viruses associated with Parkinsonism include Echovirus, PV, MeV, HSV, EBV, CMV, VZV, Borna disease virus, and WEEV [193,418]. However, in the case of PD, there are some data consistent with the notion that exposure to some viruses protects a person against subsequently developing PD [419].

We conclude that the case for any virus causing or contributing to the expression of PD is very weak, but it does not exclude several virus infections acting as cofactors in PD pathogenesis. We can also surmise that exposure to an infected colleague is highly unlikely to cause PD in the exposed person.

#### 4.2.2. Alzheimer’s Disease (AD) and Other Major Neurocognitive Disorders (MNDs)

Dementia, now referred to as major neurocognitive disorder (MND), is characterized by a progressive and persistent decline in memory and cognitive functions. It is recognized as the fifth leading global cause of death [420]. The most prevalent forms of MNDs include AD, which affects approximately 25 million individuals worldwide and accounts for 60–80% of all cases and vascular dementia (VaD) constituting around 15% of cases [421,422,423]. Familial cases of AD account for only 1–5% of the total [422]. In the case of VaD, a long list of risk factors has been documented [424], but the underlying causes of MNDs continue to impede the effective prevention of these diseases.

The majority of MNDs, including AD, are proteopathies defined by the accumulation of native proteins within the brain, which are considered key mediators of the disease pathology [425]. Surprisingly, the deposition of amyloid-beta (Aβ), an AD hallmark observed in the brain of patients, may be the brain’s physiological solution to its need to suppress the effects of an invader [426]. VaD is characterized by impaired cerebral blood flow leading to small and microscopic infarcts that collectively contribute to vascular brain damage, a key pathological feature of this syndrome [427]. Notably, this type of pathology also can be observed in other types of dementia. The amyloid and vascular theories are central frameworks that attempt to explain AD and VaD origins [428]. However, aggravating the complexity of causation and diagnosis is the frequent occurrence and coexistence of AD and VaD, making the pathological expression variable and changeable [428].

A feature playing a prominent role in the pathogenesis and progression of almost all MNDs is a persistent inflammatory response in the CNS that represents a reaction of glial cells to injurious signals that need to be repaired [429]. The root cause of chronic neural inflammatory reactions may be a persistent stimulus that eventually results in neurodegeneration [429,430], and such stimuli could include pathogens, notably viruses. In particular, when exposed to infection, microglia become more responsive to stimuli, a phenomenon known as microglial priming. The cells can serve as a major source of inflammatory cytokines, thereby exacerbating brain inflammation [431].

In fact, several named viruses have been implicated as having a potential role in the etiology of MNDs. These include members of the *Picornaviridae* family [432,433], ZIKV, HIV, MeV, IAV, HCV [434], as well as at least six members of the *Herpesviridae* family, some of which can be detected in AD patients’ brain [435]. Scrutinous observations have focused on a role for HSV-1 cerebral infection but so far without convincing evidence. The issue is made complicated by the high positive seroprevalence of HSV-1 in the general population, as well as some studies that showed that 65–75% of otherwise healthy, deceased individuals carried HSV-1 in their brain [34]. Similar findings are claimed for HHV-6, with its presence detected in the brain of up to 87.5% of people with no clinical signs of active infection or neurological disease [145]. Curiously, one study showed that around 1% of the population had the HHV-6 genome present in all nucleated cells of the body, attributed to infection and integration into progenitor germinal cells [157].

We must conclude that an abundance of circumstantial but rarely confirmed information implicates herpesviruses as involved in any neurodegenerative syndrome. However, the issue is far from settled. Conceivably, MDNs could be caused by the cumulative impact of multiple infections or exposure to a high virus dose to set off such lesions. The results of ongoing studies examining large cohorts with long follow-up periods and multiple infections might solve the dilemma. For instance, an analysis of exposure to 925 infectious diseases before dementia onset in hundreds of thousands of people concluded that dementia risk was associated with the number of episodes of hospital-treated infections [436]. The greatest risk was seen for CNS infections, but complicating the issue was the observation that extra-CNS infections also made dementia more likely. Another study examining hundreds of thousands of people and 45 viral exposures showed a strong association between viral encephalitis, or viral infections of other organs, with dementia [437]. Interestingly, the greatest likelihood of diagnosing the dementia existed in the first year after encephalitis, but a significantly elevated risk of ND was observed even 15 years after viral exposure.

Other data supporting the possible causality of viruses in dementia include post-vaccination reduceddementia risk. For instance, the nationwide shingles vaccination program against VZV in Wales has been associated with the decreased incidence of dementia in the immunized, particularly VaD and AD [438]. Interestingly, the reduction was linked to improved vascular health rather than a decreased incidence of shingles. Additionally, vaccinations against polio and influenza were associated with a 25–60% reduction in the later development of AD [439]. These findings indicate that vaccines targeting shingles, along with other vaccines for older adults, could play a role in mitigating dementia risk. However, these intriguing observations require independent verification before we rewrite the rulebook.

Assuredly, some viral infections, and particularly those related to the CNS, are linked to a sustained elevation in dementia risk. Notably, this relationship is also valid for infections outside the CNS, which implies their role in influencing brain health. Moreover, the interplay of viral infections with other factors, such as genetics, could be driving the elevated dementia incidence. Conceivably, the increased dementia risk also could be driven more by a generalized inflammatory state than by any specific or multiple infections.

#### 4.2.3. Multiple Sclerosis (MS) and Other Demyelinating Diseases

Among human demyelinating diseases with direct viral involvement, two prominent examples are progressive multifocal leukoencephalopathy (PML) and subacute sclerosing panencephalitis (SSPE). PML is caused by reactivation of the John Cunningham virus from a latent state, particularly in the individuals with immune deficiencies. The productive polyomavirus lytically infects oligodendrocytes, resulting in the formation of demyelinated lesions [440]. Patients experience neurological symptoms, such as cognitive impairment, motor weakness, and paralysis, and the disease generally follows a relentless progression, leading to death within several months [441].

Another virus-caused demyelinating disorder is SSPE, a devastating disease primarily affecting children, generally appearing 7–10 years post-measles infection. SSPE is characterized by progressive cognitive decline, behavioral changes, motor dysfunction, and seizures, ultimately leading to a fatal outcome [442,443].

A third disease where viruses are implicated, so far without solid evidence, is MS. This syndrome is a leading cause of long-term disability in young adults [444]. Usually starting at the age 20 to 40 and occurring more frequently in women, the condition is characterized by the degeneration of myelin sheaths around axons in optic nerves, as well as nerve cells in the brain and spinal cord. This leads to diffuse neurodegeneration, as the protective and trophic functions of oligodendrocytes towards neurons cease being produced. The consequence is the production of focal demyelinating plaques associated with varying levels of inflammatory cells within the brain, which in turn lead to progressive, physical and cognitive impairment [445]. The diversity of demyelination patterns observed in MS suggests variability in the mechanisms that contribute to lesion formation [446].

It is hypothesized that the pathogenesis of MS involves an autoimmune process primarily driven by myelin-reactive T cells, but this still remains an unproven concept. Antibodies targeting myelin components also have been observed in MS patients, but their role in causing lesions has not been substantiated. In addition, there is uncertainty as to the role of virus infections as causes or triggers in the development of MS [447]. Many different, named viruses have been suggested to cause MS, particularly by the late flamboyant and clever investigator Hilary Koprowski [448,449], but the strongest case that viruses might cause MS comes from studies in animal models of MS such as EAE. However, the EAE models only partially resemble MS. The most valuable study of such models uses Theiler’s virus in susceptible mouse strains, and these studies have been invaluable to identify how a virus infection could cause demyelinating lesions and set off an autoimmune reaction but also to learn how some viruses might even contribute to controlling such lesions [450]. MS is strongly suspected to represent an autoimmune lesion with myelin components as the antigen source, but the changing disease presentation typical of MS is difficult to comprehend and is not replicated by most EAE models used to study MS. Recently, based on longitudinal studies, a strong case was made that infection with the gamma herpesvirus EBV was correlated with the subsequent development of MS [451]. The study involved over 10 million active military personnel and identified 955 individuals who later or after their service developed MS.

In this comprehensive study, EBV infection was found to increase the risk of MS in the analyzed individuals by 32-fold, and EBV seroconversion occurred in MS patients who were seronegative at the beginning of the study. The effect also happened before elevation of the early marker of preclinical MS in their blood, the serum neurofilament light chain (sNfL). Moreover, the risk of developing MS turned out to be extremely low in those individuals who had not been infected with EBV. These studies were persuasive, but not entirely convincing, and obviously have not been duplicated by an independent study. In addition, and of interest, those who contracted EBV-caused infectious mononucleosis also had an increased risk of developing MS [452], and the presence of antibodies against Epstein–Barr nuclear antigens acted as a strong predictor of MS risk up to 20 years after their appearance [453]. Nevertheless, up to 90% of individuals worldwide are infected with EBV, but only a small minority develop MS, indicating that many other factors are involved in development of the disease. The studies implicating EBV as a cause of MS make a strong case, but the jury is still deliberating. Moreover, were EBV to be a significant causative factor, no way currently exists to prevent the infection or to modulate its infection process.

#### 4.2.4. Amyotrophic Lateral Sclerosis (ALS)

One tragic, invariably lethal, rare, neurodegenerative disorder of the nervous system that possibly involves viruses is ALS. During ALS progression, the degeneration and rapid loss of motor neurons occur in the motor cortex, brainstem, and anterior horns of the spinal cord [454]. This motor neuron loss results in muscle atrophy with progressive muscle weakness that leads to paralysis, respiratory failure, and, ultimately, death, which typically occurs within 3 to 5 years following onset [455].

Interestingly, up to 50% of ALS patients experience varying levels of cognitive impairment, indicating the additional potential involvement of systems beyond the motor pathways in the disease pathogenesis. ALS can present in either familial or sporadic forms, the latter accounting for about 90–95% of cases [456,457]. The evidence that some virus acts as a cofactor in the cause or expression of ALS remains a debated possibility. The favored candidates for involvement are enteroviruses [4]. These agents are advocated to cause RNA-processing abnormalities, disrupted nucleocytoplasmic transport and consequent protein aggregation, neuroinflammation, deficits in protein quality control mechanisms, as well as the TAR DNA-binding protein 43 (TDP-43) expression observed in motor neurons and glial cells of around 97% ALS patients [458]. The involvement of PV, coxsackievirus, echovirus, enterovirus-A71 (EV-A71), as well as enterovirus-D68, in the development of ALS, also has been hypothesized [457]. However, the causative role of a known virus remains debatable, which is fortunate since it means that ALS patients are not an infectious danger to others. Investigating the possible role of viruses in ALS is also made problematic by the lack of good animal models to study the issue.

#### 4.2.5. Human Endogenous Retroviruses and Their Association with Neurodegenerative Syndromes

Human endogenous retroviruses (HERVs) constitute around 8% of the human genome and are dormant remnants of exogenous retroviruses that incorporated into germline DNA in the distant past and are now vertically transmitted [459]. In addition to their high copy numbers contributing to genomic variation [460,461], the set of HERVs integrated at identified genomic sites can differ among individuals [461,462]. Most open reading frames of HERVs are mutated, rendering them incapable of producing functional proteins or virions, or they are silenced through epigenetic mechanisms [463,464]. Nevertheless, endogenous retroviruses can still influence nearby genes by serving as alternative promoters, or by modifying chromatin structure [465]. Additionally, aging may promote the loss of heterochromatin, and this could result in the transcriptional activation of these normally silenced elements [466]. Moreover, infections with several human viruses, which include HIV-1, HTLV-1, HBV, IAV, and some herpes viruses, can act as triggers of HERV expression [464]. Under favorable physiological or pathological conditions, some endogenous retroviruses may contribute to the onset of neurodegenerative disorders and other syndromes (see Table 8).

However, the mechanistic circumstance that precipitates such events is poorly understood. Nevertheless, evidence continues to accumulate that links endogenous retroviruses to MS, ALS, and other chronic neurodegenerative syndromes [471]. Moreover, endogenous retroviruses are known to influence several aspects of innate immune responsiveness that, in turn, could affect the pathogenesis of neurodegenerative disorders. Furthermore, the increased expression of HERV genes or levels of antibodies against particular HERV peptide fragments could serve as possible biomarkers in patients with MS or ALS [469,473]. In fact, clinical trials have already been initiated in treating diseases where HERVs are implicated [5]. Moreover, in this age where removing or modifying HERVs is becoming feasible, the prospect of the management of chronic neural lesions could become realistic. The topic of how different endogenous retroviruses could play a role in the causation and outcome of various neurological and other chronic diseases, as well as in aging, has been covered in detail in other reviews [5,464,474,475].

## 5. Conclusions

The brain is the body’s sanctuary. This delicate and essential organ is highly protected against infectious violators since, when this happens, usually a high price must be paid. However, viral invasions do happen, and we describe the circumstances wherein this occurs. We then portray the acute consequences of invasions by several known viruses, after which we discuss the many instances of usually chronic neurodegenerative syndromes where viral involvement is advocated but most often not substantiated. However, with these chronic diseases, which include some common named syndromes such as AD, PD and MS, rarely, if ever, is a single named virus the sole cause of damage to the brain’s function. Instead, viruses are but one of many cofactors that can contribute to the syndromes.

Few viruses have the properties that permit their access to the CNS. To get there from an initial extraneural infection site, they must either transport along nerves that enter the CNS or cross membrane barriers that act as significant obstacles to the passage. Those able to achieve CNS access express several strategies that allow passage, and these activities could be inhibited by therapeutic procedures that strengthen the barrier function. This strategy should be achievable with anti-inflammatory drugs in situations where barrier permeability is increased by inflammatory factors derived from the virus or the host itself. In other circumstances, vaccines against the causative viruses can be effective, but, except for Rabies virus with its often extraordinarily long incubation time after infection before it expresses lesions and perhaps shingles, vaccines must be used prior to infection to be effective against neural consequences. Some prophylactic vaccines have been extraordinarily efficacious, most notable those against polioviruses, but few viral neuropathogens are currently controllable with vaccines. We expect this situation to change when more is known about the molecular events involved during infection and disease along with the application of new developments in vaccine design that can target critical events in viral pathogenesis and are less toxic. Accordingly, effective vaccines would be useful to minimize the impact of Nipah viral infection, as well as some human flavivirus infections that currently lack vaccines. Vaccines are used effectively to control several encephalitides of veterinary relevance; for example, VEE and an attenuated VEE vaccine are used by special groups, such as the US military, and they have side effects that the general public would not accept.

Another control measure to protect the brain would be to use antiviral drugs and perhaps biologicals. Alas, this approach is currently used against very few viruses that infect the brain. Antivirals are valuable to counteract the severe consequences of HSV and HIV infections, although, in most instances, they do not succeed in removing the virus from the brain or some other locations. Other neural infections potentially controllable by antiviral therapy include infections that are caused by some of the flaviviruses and togaviruses, since effective antivirals are used against similar viruses that mainly cause non-neural diseases. Most notable are the highly effective antivirals used against the hepatitis C flavivirus.

While it seems fair to expect that the future will see therapies and vaccines that effectively manage some acute viral neural diseases, controlling chronic ND syndromes by targeting the putative viral involvement will remain far more problematic. Thus, although viruses are advocated to cause, or at least contribute, to the development and course of several major ND diseases, which include AD, MS, PD and several others, controlling them by targeting viruses seems implausible. Moreover, any role for a virus in those major ND diseases is likely to be as a cofactor amongst many others. Thus, targeting viruses to control such diseases will be futile. With some chronic NDs, such as ALS, the evidence for viral involvement is stronger, but controlling ALS by therapies that target a virus are ineffective at present. Nevertheless, the good news with most if not all chronic ND syndromes is that the affected person is unlikely to be an infectious source of danger to others. With some chronic NDs, a case linking them to a named virus, such as EBV with MS, has strong circumstantial support, but, even if substantiated, effective vaccines or antivirals currently are lacking to control the possible viral cause. We can conclude that much has been learned, but more needs to be known before we solve the problems that viruses cause when they access the brain. Stay tuned.

## Figures and Tables

**Table 1:** Neuronal pathways to CNS invasion used by RABV and HSV-1.

	RABV	HSV-1
Cells through which the virus enters the host’s body	Muscle cells	Epithelial cells
Method of entry into the peripheral neuron	Receptor-dependent clathrin-mediated endocytosis [39,62]	Fusion of virus lipid envelope with host cell membrane [63]
Viral proteins responsible for neuron entry	RABV G—the only protein exposed on the surface of the viral envelope that can bind to various receptors [64,65]	At least 12 different surface glycoproteins bind to more than one receptor [66]
Type of neuron used on the way to the CNS	Motor neuron	Sensory neuron
Mechanism of virus transport in a neuron	Fast dynein- and kinesin-mediated transport on microtubules in endosomes [67]	Dynein, dynactin, and kinesin-1-mediated transport on microtubules of the non-enveloped capsid [68]
Reaching the brain and the fate of the host	The virus always reaches the brain cells, ultimately causing the host’s death	If the virus reaches the brain cells, it may cause severe acute, potentially fatal encephalomyelitis, but this rarely happens
Ability to persist in the brain without causing the host’s death	No	Yes, in a latent form in the brainstem, as well as in other brain regions until the host’s natural death (not explained how it reaches them)
Main factor(s) determining tissue and cell tropism	Multiple—high level of receptors on the host cell surface, lower protective immune response of neurons, low immune surveillance over cells, others	The occurrence of specific receptors on host cells’ surface
Inflammatory response in the brain during acute infection	Specific suppression of the host immunity through diverse mechanisms [46]	Brain parenchyma inflammation is accompanied by an inflammatory response that may become exaggerated and further increases brain injury [69]

**Table 2:** Viral pathways of neuroinvasion.

Possible Hematogenous Neuroinvasion Through Brain Barriers	Viruses
1. Transcellular pathway with none or minimal barrier damage	*Flaviviridae*: WNV, JEV, TBEV, ZIKV; *Togaviridae*: VEEV, WEEV; *Retroviridae*: HIV-1
2.1. Infection with general damage to barrier cells	*Flaviviridae*, *Paramyxoviridae*: HeV, NiV
2.2. Paracellular pathway with direct impairment of intercellular junctions of barrier cells	*Flaviviridae*: WNV, JEV, DENV *Picornaviridae*: Enterovirus A71
2.3. Barrier damage by inflammatory mediators	*Coronaviridae*: SARS-CoV-2
3. Trojan horse mechanism	*Flaviviridae*: JEV, WNV, ZIKV *Retroviridae*: HIV-1, Visna virus

**Table 3:** Viruses responsible for neuropathology and clinical outcomes in humans.

	Virus	Common Way of Entry to the CNS	Frequent Clinical Presentation	Outcome and Viral Persistence	Infected CNS Cells
Family: *Herpesviridae*	HSV-1, HSV-2	Anterograde transport from TG (HSV-1) or sacral dorsal ganglion (HSV-2) by sensory neuron projections [137], via olfactory pathway [138].	Severe acute necrotizing encephalitis (HSV-1), meningitis (HSV-2) [139], or asymptomatic presence—accumulating data from the autopsy of people deceased without neurological illnesses (2–100%) [140].	Encephalitis often leads to lifelong brain damage or death. Virus undergoes latency and persists permanently within neural tissue.	Neurons, astrocytes, microglia, oligodendrocytes [141].
	VZV	Anterograde transport by neuron projections from dorsal root ganglia and cranial nerve ganglia.	Meningitis or encephalitis often without concurrent zoster rash [142,143] or asymptomatic presence—data from the autopsy of people with no evidence of neurological disease (1–27.5%) [144,145].	Resolution of symptoms with or without neurologic sequelae, encephalitis can result in fatal outcome. Virus is suspected to persist in the CNS, as it establishes latency in neurons.	Neurons, astrocytes [146], oligodendrocytes [147].
	EBV	Via infected lymphocytes.	Meningitis, encephalitis, and CNS-lymphoma.	Resolution of symptoms without or with long-term consequences (depending on the host immune status and other factors [148]), may end with death. Establishes lifelong infection of the PNS and/or CNS, EBV-infected cells, free virus, and gene products can be found in the CNS [149].	Neurons [150], astrocytes, microglia [151].
	CMV	Presumably via infected monocytes [132].	Meningitis, encephalitis (particularly in immunosuppressed individuals).	In immune-naïve and immunocompromised persons can cause CNS complications or result in fatal outcome [152]. Routinely establishes lifelong infection of the PNS and/or CNS [149].	Neurons (debated), astrocytes, microglia, oligodendrocytes, neural progenitor cells [153].
	HHV-6A, HHV-6B	Via olfactory pathway [154], infected lymphocytes, or optic tract [155].	Encephalitis or asymptomatic presence—data from autopsies of individuals who passed away without neurological disorders (19–87.5%) [145,155,156].	Encephalitis often has devastating sequelae or may cause death [157]. Viruses establish the lifelong infection of the PNS and/or CNS [149].	Neurons, astrocytes [158], microglia [159], oligodendrocytes [160].
Family: *Picornaviridae*	Poliovirus	Via the BBB and motor neurons [72].	Paralytic poliomyelitis.	Severe and irreversible damage of motor neurons, among those paralyzed, 5–10% die [161]. It is suggested that the virus may persist in surviving patients [162].	Neurons [72].
	Enterovirus-A71	Hematogenous way—transcellular pathway and/or infection of endothelial cells [163].	Meningitis, encephalitis, polio-like syndrome, encephalomyelitis (the risk may be associated with younger age) [164].	CNS involvement may entail severe sequelae, death is rarely reported [165]. The mechanisms and extent of EV-A71 persistence in the human CNS is unknown [166].	Neurons, astrocytes [167], microglia [168].
Family: *Orthomyxoviridae*	Subtypes of influenza A virus	Presumably via olfactory nerve pathway [169,170,171], cranial nerves [172], via BBB endothelial cells [173].	Acute encephalitis or influenza-associated encephalopathy.	Severe onset, often resulting in significant morbidity and high mortality within a matter of days [173].	Neurons [174], astrocytes [175].
Family: *Paramyxoviridae*	Mumps virus (MuV)	Through the choroid plexus—virus infects choroidal and ependymal epithelial cells [176], on mononuclear cells transiting the BBB during viremia.	Meningitis.	Usually mild or asymptomatic disease with often complete recovery [177]. Symptomatic CNS infection is less common and rarely fatal [178]. In rare instances, the virus is believed to persist in the CNS [179,180].	Neurons [181].
	Measles virus	Hematogenous way—infection of brain endothelial cells [182], in infected CNS infiltrating macrophages [183].	Primary encephalitis, postinfectious encephalomyelitis, SSPE.	All diseases can lead to severe and permanent brain injury [184], SSPE is invariably fatal. Virus may persist in neurons [185].	Neurons, astrocytes, oligodendrocytes [183].
	Henipaviruses (HeV, NiV)	Directly from the nasal mucosa and in a hematogenous way [186].	Febrile illness or encephalitis.	The disease may be fatal, survivors may experience severe neurological sequelae [187].	Endothelial cells of the CNS microvasculature, neurons [188], astrocytes, oligodendrocytes [189].
Family: *Rhabdoviridae*	RABV	Retrograde axonal transport.	Progressive rabies encephalitis.	Invariably fatal outcome.	Neurons, astrocytes (field strains) [53].
Family: *Flaviviridae*	Encephalitic flaviviruses	Along the peripheral nervous system and axonal transport, hematogenous route—the infection of choroid plexus cells [190], the infection of BBB cells without cytopathic effect, with the downregulation of junction proteins, or crossing the BBB in leukocytes [83].	Severe encephalitides and other neurologic syndromes.	Encephalitides are often fatal or may lead to transient or permanent neurological deficits [96]. Viruses may persist in the CNS.	Neurons, astrocytes, microglia, oligodendrocytes (please ref. to Table 5).
Family: *Togaviridae*	VEEV, WEEV, EEEV	Anterograde transport along peripheral nerves, olfactory route [191], hematogenous route—transcytosis across intact BBB [89].	Mild, febrile illness to severe encephalitis.	Encephalitides have 1–75% fatality rate, depending on the virus strain [89]. Virus replication and/or genomic RNA may persist in the CNS [192].	Neurons, astrocytes, microglia [193].
Family: *Retroviridae*	HIV	In HIV-infected monocytes.	HAND.	Cognitive, motor and behavioral abnormalities [194]. Virus persists in the CNS.	Microglia, astrocytes [195].
Family: *Coronaviridae*	SARS-CoV-2	Via olfactory epithelium, via optic nerve, hematogenous pathway—by infection of brain endothelial cells [196].	Encephalopathy (in most critically ill COVID-19 patients), PASC.	With serious or long-term neurological dysfunctions and deficits.	Neurons, astrocytes, choroid plexus epithelial cells [197].

**Table 4:** Encephalitis and other neurological syndromes associated with selected neurotropic flavivirus infections.

	JEV	WNV	TBEV	ZIKV
Disease transmission	Mosquito bite	Mosquito bite	Tick bite, consumption of unpasteurized milk from infected ruminants (1% of infections) [202]	Mosquito bite, placental transmission, sexual transmission [203]
Geographical regions of most encephalitis cases	Asia, Australia, Western Pacific [204]	Europe and North America [82]	Central/Northern Europe and North-Eastern Asia [205]	Africa, South-East Asia, Micronesia, French Polynesia, both Americas [206]
Most frequently encephalitis-bearing patients	Children	Elderly or inmunocompromised individuals [207]	Adults	Newborns
Vaccines available	Yes	No	Yes	No
% of people infected with the virus who develop encephalitis	0.1–1% [208]	0.67% [209]	2–25% [210]	Unknown
Number of encephalitis cases annually	~100,000 [211]	Few thousand [212]	10,000–15,000 [213]	Unknown in newborns, very few cases of adult people [214]
Encephalitis mortality rate	20–30% [215]	Over 10% [216], 10–20% for WNV meningitis [217]	1–40% [218]— in severe infections, TBE manifests as meningoencephalitis with significant mortality rate [219]	Unknown
Other neurological syndromes associated with infection	Meningitis [220], poliomyelitis-like flaccid paralysis [221], Parkinsonian syndrome [222], neuropsychiatric sequelae, cognitive and language impairment [223], GBS [224,225]	Meningitis, poliomyelitis [226], GBS [227], acute flaccid paralysis [228], movement disorders, cognitive difficulties [229], neuropsychiatric sequelae [230,231]	Meningitis, GBS [232], permanent paresis, postencephalitic syndrome [210], epilepsy, lateral sclerosis, dispersed sclerosis, Parkinson-like disease, mental deterioration leading to severe dementia and/or death [233]	Microcephaly and other congenital malformations in newborns, GBS—predominate presentaion in adults [234], encephalopathy, meningitis, myelitis, seizures [214]

**Table 5:** Non-neuronal cells infected by flaviviruses in the human CNS.

**Flavivirus**	Non-neuronal CNS cells that can be infected
**JEV**	astrocytes [82], microglia, BMECs [240], pericytes [241], neural progenitor stem cells [81]
**WNV**	astrocytes [242], oligodendrocytes [243], BMECs [244], pericytes [245]
**TBEV**	astrocytes, oligodendrocytes [205], microglia [246], BMECs, pericytes [247]
**ZIKV**	astrocytes, oligodendrocytes [248], microglia, BMECs [249], pericytes [190], neural progenitor cells [250], glial progenitor cells [251]

**Table 6:** Autoimmune conditions and related flavivirus infections.

Autoimmune Condition	Definition	Onset Time	Flaviviral Infections Associated with Autoimmune Condition
Autoimmune encephalitis, AIE	Acute to subacute progressive inflammation of the brain, associated with the presence of antibodies against self-antigens expressed in the CNS, often neuronal proteins [262].	Can develop early or during onset of encephalitis, usually during recovery from viral infection or several weeks later [263].	JEV, WNV [264,265]
Acute disseminated encephalomy-elitis, ADEM	Acute post-infectious progressive inflammatory disorder characterized by brain and spinal cord demyelination and other types of neural damage [266] can also occur post-vaccination.	Develops during or within days to weeks after CNS infections or after illnesses with CNS involvement.	DENV, ZIKV [237,267,268,269]. Immunization against JEV [270].
Guillain–Barre Syndrome, GBS	Post-infectious neuropathy resulting from the autoimmune destruction of nerves in the PNS [271].	Typically begins within a few days to several weeks after a viral infection.	DENV, WNV, JEV, and ZIKV [224,227,272,273].

**Table 7:** Neurotoxic effects of HIV proteins in the brain.

Direct injury of neurons	gp120, gp41, Tat, Vpr [300,301,302,303]
Induction of pro-inflammatory cytokine expression and release; glial and myeloid cell activation	Tat, gp120, Nef, Vpr [122,304,305,306,307,308,309,310,311,312]
Suppression of NF-κB-elicited antiviral immune responses	Vpu [313,314]
Induction of adhesion molecules expression and mediation of adhesion molecules’ release	Tat, gp120, Nef [315,316,317,318]
Regulation of protein stability through modulation of ubiquitination	Vif, Vpu, Vpx, Vpr, Nef [319,320]
Promotion of apoptosis	gp120, Tat, Vpu, Nef [319,321,322]
Inhibition of apoptosis	Tat, Vpr, Nef, Vif [323,324]
Alteration of intracellular ionic homeostasis	Tat, gp120, Nef, Vpr, Vpu, gp41 [325,326,327,328,329,330,331,332]
Disruption of cell–cell communication	Tat, Nef, gp120 [333,334,335,336,337]
Disruption of metabolic pathways	gp120, Tat, Vpr [338,339,340,341,342]
Disruption of DNA repair	Vpr [343,344,345]
Disruption of neurotransmitter signaling	[346]
Induction of ROS and RNS production	Tat, Vpr, Vpu, gp120, gp41, Nef [347,348,349,350]
Interaction with Aβ and/or Tau resulting in their accumulation (disruption of processing, localization, and phagocytosis by microglial cells)	Tat, gp120, Vpr, Nef [351,352,353]
Modulation of infected T cell activation status	gp120, Env, Nef, Tat, Vpr [354,355,356,357,358]

**Table 8:** Endogenous retroviruses implicated in neurodegenerative disorders.

Neurodegenerative Disorder	HERVs Implicated in Disease
MS	HERV-W, HERV-K, HERV-H, HERV-E, HERV-_15_ [467,468].
ALS	HERV-K, HERV-W [469]
AD	HERV-H, HERV-K, HERV-L, HERV-W [5,470]
progressive supranuclear palsy	HERV-H, HERV-K, HERV-L [470]
FTD	HERV-K [471]
PD	HERV-K [5,472]
